# Morphological and Immunohistochemical Changes in Progressive Postmortem Autolysis of the Murine Brain

**DOI:** 10.3390/ani14243676

**Published:** 2024-12-20

**Authors:** Francesca Parisi, Sara Degl’Innocenti, Çağla Aytaş, Andrea Pirone, Carlo Cantile

**Affiliations:** 1 Department of Veterinary Sciences, University of Pisa, Viale delle Piagge 2, 56124 Pisa, Italy; francesca.parisi@unipi.it (F.P.); cagla.aytas@phd.unipi.it (Ç.A.); andrea.pirone@unipi.it (A.P.); 2 GLP Test Facility, San Raffaele Telethon Institute for Gene Therapy (SR-Tiget), IRCCS San Raffaele Scientific Institute, Via Olgettina 60, 20132 Milan, Italy

**Keywords:** brain, C57BL/6J mouse, autolysis, postmortem interval, forensic veterinary pathology, immunohistochemistry

## Abstract

In this time series study, the temporal sequences of postmortem changes in brains kept at different temperatures were investigated in different areas of mouse brains. Fixation of tissues kept at different storage temperatures (4 °C, 22 °C, 37 °C) was delayed for four time points (24, 120, 168, 336 h). Histological and immunohistochemical investigations were carried out to determine how postmortem autolysis may affect the cellular morphology and the expression of neural cell epitopes. Results showed that the autolytic changes started earlier in brains at 22 °C and 37 °C and in the grey matter compared to the white matter, with the cerebellum and hippocampus showing the earliest postmortem changes. The cellular antigens were differently affected by the autolytic process overtime: NeuN and Olig2 immunoreactivity was gradually lost at the nuclear site and diffused into the cytoplasm; increased background staining was observed with SMI-32; GFAP showed an increase in immunolabeling, whereas 2F11 immunoreactivity decreased. This study suggests that the morphological analysis and immunohistochemical investigation of the brain tissue could be satisfactorily applied to forensic cases, providing useful data for the estimation of the postmortem interval.

## Simple Summary

The study investigated postmortem changes in mouse brains stored at different temperatures and examined at different time points through histological and immunohistochemical analyses. The findings revealed earlier autolytic changes in brains stored at higher temperatures and in the grey matter compared to the white matter, particularly in the cerebellum and hippocampus. Over time, NeuN, Olig2, and 2F11 immunoreactivity decreased, SMI-32 showed increased background staining, and GFAP exhibited increased immunolabeling. The study suggests that these analyses could be helpful in estimating the postmortem interval in forensic cases.

## 1. Introduction

Postmortem autolysis is a common occurrence after death, marking the beginning of decomposition. The cessation of metabolic processes and the deprivation of oxygen trigger a series of events that result in cell death and self-digestion (autolysis) [1,2]. This process is primarily caused by the release of cathepsins and other hydrolases due to lysosome and peroxisome membrane permeabilization [3]. As oxygen levels decrease postmortem, anaerobic conditions develop, with anaerobic glycolysis providing energy for basic cellular functions. Waste products like carbon dioxide and lactate are released, causing a drop in cellular pH until the membrane loses its normal permeability, leading to cytoplasmic membrane disruption [4]. The term oncotic necrosis (or oncosis) is also proposed to describe the postmortem cell death pathway induced by the depletion of cellular adenosine triphosphate (ATP) [5]. Putrefaction then occurs as microorganisms, including bacteria, fungi, and protozoa from the gastrointestinal tract, degrade tissues, resulting in complete tissue decomposition.

Although autolysis and putrefaction can be distinguished on a molecular level, both processes can occur simultaneously to some degree in a decaying corpse [6]. However, autolytic changes are typically only detectable microscopically, whereas most postmortem changes resulting from putrefaction are visible macroscopically, and the decomposition byproducts may appear as gas, fluid, or salt form.

The histological identification of autolytic changes in various organs at varying postmortem intervals can enable forensic pathologists to more precisely determine the postmortem interval (PMI), thereby offering a clearer timeframe for the occurrence of death. While autolysis generally occurs simultaneously across the body, distinct cell types may exhibit varied responses to this process, influenced by their individual resistance to hypoxic conditions [2,7,8,9,10,11,12]. Organs such as the liver and brain, which are involved in the generation of high levels of ATP and membrane transport, are notably more prone to autolysis compared to other tissues [4]. The heart and kidney are moderately susceptible to the autolytic process in contrast to fibroblasts, which, due to their relatively scant lysosomes and lower levels of hydrolytic enzymes, exhibit significantly slower autolysis [13,14].

Given that the brain is regarded as more prone to autolysis than other organs [4], examination of decomposed brain tissue is infrequently conducted during forensic autopsy, often resulting in this aspect being overlooked in forensic neuropathology. Despite this, the histological and immunohistochemical examination of the brain, including cases of decomposition, has proven to be beneficial in legal contexts, often indicating that the histological features of the nervous tissue are substantially more informative than what might be anticipated from the overall gross condition of the brain [15].

A notable characteristic of the brain is its encasement within the cranium, offering a protective barrier against external factors, scavenger animal attacks, and atmospheric humidity, particularly in cases of death occurring in open environments. Delayed examination of human brain tissue has been shown to yield useful results, as demonstrated by achieving a diagnosis of Alzheimer’s disease at autopsy 2 months [16] and 20 months [17] after death.

Numerous studies focused on CNS autolytic changes have been conducted for many years, but they were mostly restricted to some specific areas, neural cells, or cell components (i.e., dendrites, axons, synapses) [18,19,20,21]. Histological and histochemical staining techniques (i.e., hematoxylin and eosin, Nissl stain) and electron microscopy were the primary methods used for investigation, whereas immunohistochemical analysis has only recently been considered for PMI estimation [19,20,22]. Moreover, brains were usually kept at room temperature (22–23 °C) [19,20,23], and in most studies, an experimental postmortem period of a maximum of 24–48 h was adopted [19,23].

Research focused on understanding common postmortem autolytic changes and the factors that influence them has been ongoing for years, as these changes can help estimate PMI. However, a more comprehensive experimental animal model is needed to study morphological and immunohistochemical autolytic changes in the CNS in a diagnostic context. Our study was specifically designed to fill this gap by conducting an in-depth analysis, considering extended postmortem fixation delays and tissue preservation over a wide temperature range.

## 2. Materials and Methods

### 2.1. Animals

A total of 94 C57BL/6J mice, 4- to 6-month-old, were used for this study. Animals were euthanized as part of the planned sacrifices for the maintenance of the colony numbers at the laboratory animal facility of the Consiglio Nazionale delle Ricerche (CNR) in Pisa (Italy) and collected straight after death. They weighed, on average, 32 ± 10 g and both males and females were randomly included. None of the mice were noted to display clinical or gross signs of neurological disease.

### 2.2. Experimental Design

The mice were divided into 12 experimental and 2 control groups, consisting of 7 and 5 animals per group, respectively. All mice were sedated using 80 mg/kg of Zoletil^®^ (Virbac S.r.l., Milan, Italy) and 10 mg/kg of Rompun^®^ (Elanco Italia S.p.A., Milan, Italy). 0.1 mL of heparin was administered intraperitoneally to 5 mice, which were then transcardially perfused with cold phosphate-buffered saline followed by 4% paraformaldehyde in PBS. The brains were removed following a standard procedure [24] and put in 10% neutral buffered formalin (control group of perfusion-fixed brains, pT0). All the remaining mice, after sedation, were euthanized using an overdose of isoflurane. After euthanasia, 5 mice for each study group were decapitated at the level of C3. The brains of another group of 5 mice were removed immediately and fixed by immersion in 10% neutral buffered formalin (control group of fixed brains, fT0). Fixation of the brains of the experimental groups was delayed for varying times (T1 = 24, T2 = 120, T3 = 168, T4 = 336 h), maintaining the heads at different storage temperatures (4 °C, 22 °C, 37 °C). To determine whether there was any difference between preserving the entire body or just the separate head, 2 mice from each experimental group were preserved whole. Macroscopical examination of the brains was carried out after fixation, followed by histopathological and immunohistochemical analyses.

### 2.3. Histopathology

Transverse sections of the fixed brains were embedded in paraffin wax, and 5 µm sections were routinely processed for histology according to the revised procedures of the National Toxicology Program [25]. A morphological and immunohistochemical study was performed to evaluate both grey (GM) and white matter (WM). The cerebral and cerebellar cortices, thalamus, hippocampus, and cerebellar nuclei were selected as representative GM areas. The corpus callosum, internal capsule, and cerebellar WM were evaluated as WM structures. Other brain structures, such as leptomeninges, choroid plexus, ependyma, and neuroparenchymal vessels, were also evaluated.

For the morphological study, brain sections were stained with Hematoxylin and Eosin (HE), Nissl staining for GM, and Luxol Fast Blue (LFB) for WM. Five non-overlapping microscopic fields at high power magnification (400×) (1.185 mm^2^) were evaluated for both the morphological and immunohistochemical examination in all the selected neuroanatomic areas. A 4-tier semiquantitative scoring system, ranging from 0 (severe) to 3 (absent), based on the presence of specific autolytic changes, was assigned to GM and WM areas in sections stained with HE, Nissl staining, and LFB (Table 1).

### 2.4. Immunohistochemistry

Nerve cell changes were examined by immunohistochemistry (IHC) at all time points using antibodies against neuronal nuclear protein (NeuN), non-phosphorylated neurofilaments (SMI-32), phosphorylated neurofilaments (2F11), glial fibrillary acidic protein (GFAP), and oligodendrocyte transcription factor 2 (Olig2). The list of the primary antibodies, as well as the dilution and the antigen retrieval method used, are reported in Table 2.

Heat-induced epitope retrieval using a pressure cooker was performed for all markers except for GFAP. Primary antibodies were incubated overnight at 4 °C. Rabbit polyclonal antibodies immunoreactivity was detected by the avidin-biotin-peroxidase complex method (Vectastain^®^ Elite ABC-Peroxidase kit; Vector Laboratories, Inc., Burlingame, CA, USA), whereas EnVision+ labeled polymer HRP anti-mouse was used for mouse monoclonal antibodies. The 3,3′-diaminobenzidine (DAB) was used as chromogen in all studied antibodies.

Brain sections of formalin-fixed and perfused control mice (fT0 and pT0) were used as control cases. Negative controls were obtained by omitting the primary antibody. Positive and negative control slides were run with each batch of slides.

Immunohistochemistry was evaluated using a modified 4-tier classification system [26], as shown in Table 3. This system combined both immunolabeling specificity and intensity, respectively defined as the appropriate pattern of antigen immunolocalization and the amount of chromogen observed compared to control tissues. In detail, for each marker, one experimental group at a time was directly compared to the control groups (taken together) and evaluated both for specificity and intensity by the pathologists involved in the study (FP, SDI, ÇA, CC). Additionally, a quantitative analysis of NeuN immunolabeled neurons was manually performed using NIS-Elements BR 4.00 (Nikon Instruments S.p.A., Firenze, Italy). Five non-overlapping microscopic fields at high power magnification (400×) were evaluated, covering a total area of 1.185 mm^2^ for each anatomical area, and the number of immunolabeled cells was registered.

### 2.5. Statistical Analysis

The numerical reduction of NeuN immunolabeled neurons in the cerebral and cerebellar cortex, thalamus, and hippocampus was analyzed in relation to storage temperatures and delayed fixation time; fT0 and pT0 groups were also compared. Only neurons with clearly distinguishable immunolabeled nuclei were counted, whereas neurons showing cytoplasmic immunolabeling were not considered. The statistical analysis was performed with JASP (version 0.19.1). Continuous variables were expressed as the median and interquartile range as the data were not normally distributed (Shapiro-Wilk test). Data were analyzed by the Kruskal–Wallis test, followed by Dunn’s multiple comparison test with Bonferroni correction. Statistical analysis for other markers was not performed because their evaluation was primarily descriptive, without any calculating measures of inter-observer variability.

## 3. Results

### 3.1. Gross Examination

Grossly, all the brains did not show remarkable features of decomposition and putrefaction at all time points. A mild reduction of brain tissue consistency was present in brains kept at 22 °C and 37 °C at T3 and T4, accompanied by a stronger pink discoloration of the nervous tissue. Other brain structures (i.e., meninges, main arteries, choroid plexus) were clearly recognized, allowing for a macroscopical examination. No significant differences were found between brains removed from decapitated heads and entire corpses.

### 3.2. Histopathology

**Figure 1:**
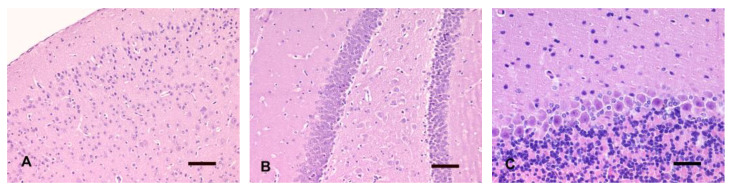
Representative sections of a fT0 brain. (**A**) Cerebral cortex. (**B**) Hippocampus. HE, bar = 100 µm. (**C**) Cerebellar cortex. HE, bar = 50 µm.

The histomorphological features of the nervous tissue did not differ from decapitated heads and entire corpses, as well as within the control groups (fT0 and pT0). The tissue morphology considered normal is depicted in Figure 1.

In mice belonging to the same study group, autolytic changes in the different brain areas homogeneously affected the neuroparenchyma of the evaluated area, so one single score was assigned to every area at every time point. Where the brain score was different, it was reported. Histopathological scores are summarized in Table 4.

#### 3.2.1. Brains Kept at 4 °C

##### T1 (24 h)

**Figure 2:**
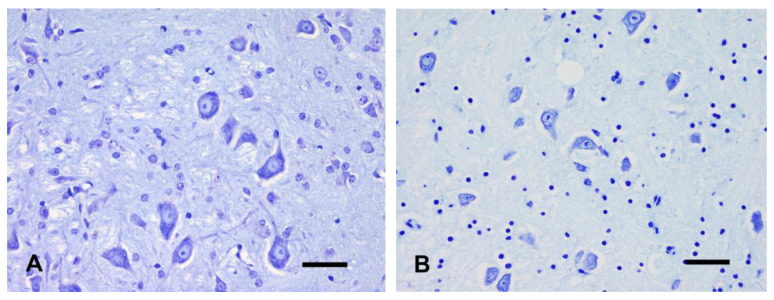

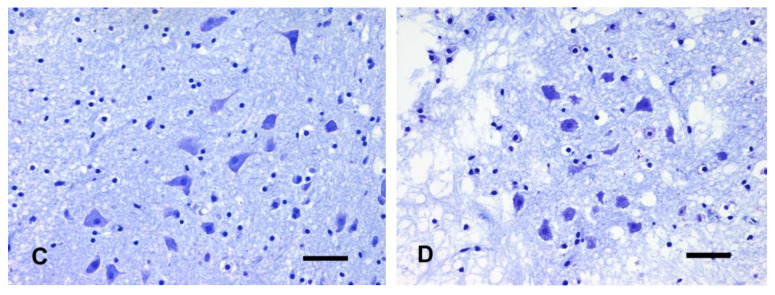
Brains kept at 4 °C, cerebellar nuclei. (**A**) T1. Nissl substance is recognizable within the cytoplasm of most neurons. Neuronal nuclei and nucleoli, glial cells, and neuropil are normal. (**B**) T2. Pericellular clear halos and slight cell shrinkage. (**C**) T3. Moderate neuronal shrinkage and nuclear pyknosis. (**D**) T4. Severe neuronal shrinkage, nuclear pyknosis and neuropil vacuolization. Nissl staining, bar = 50 µm.

No autolytic changes were observed in the different brain areas and cell types of both GM and WM of all brains (Figure 2A).

##### T2 (120 h)

Perivascular and perineuronal clear halos were evident in both the forebrain and cerebellum (Figure 2B). Cerebrocortical neurons showed only slight changes characterized by nuclear hyperchromasia and cytoplasmic shrinkage, prevalently involving large pyramidal neurons. Similar changes were also observed in the thalamus. Moderate vacuolation of the dentate gyrus was evident, accompanied by almost 50% of hippocampal neurons displaying nuclear hyperchromasia and scattered cytoplasmic vacuolation. In the cerebellum, the granule cells showed scattered nuclear pyknosis, whereas only a few Purkinje cells showed cytoplasmic microvacuolation and shrinkage, frequently accompanied by nuclear hyperchromasia. No significant changes were observed in WM areas.

##### T3 (168 h)

Nuclear pyknosis in less than half of neurons was observed in the cerebral cortex and thalamus. Marked vacuolation of the dentate gyrus was evident. Seventy percent of neurons of the dentate gyrus showed nuclear pyknosis with irregular nuclear profiles, and karyorrhexis was detected in the remaining cells. Almost all cerebellar granule cells appeared pyknotic, and a few displayed nuclear karyorrhexis. Nuclear hyperchromasia was observed in the cerebellar nuclei (Figure 2C) and in less than half of the Purkinje cells, accompanied by nuclear pyknosis and hypereosinophilic cytoplasm in a minimal percentage of cells. A mild dilation of myelin sheath was observed in forebrain WM, whereas WM changes in the cerebellum were moderate.

##### T4 (336 h)

No changes were observed in the cerebral cortex and thalamus compared to T3, except for a slight increase of neurons displaying nuclear pyknosis. Only a few cortical neurons showed irregular nuclear profile, but no evident karyorrhexis was observed. Similarly, there were no morphological differences compared to T3 in the cerebellar cortex. The hippocampus showed the most significant progression of autolytic changes. Neurons of all hippocampal regions and cerebellar nuclei displayed cytoplasmic hypereosinophilia, loss of Nissl substance, nuclear pyknosis, and karyorrhexis (Figure 2D). Moderate dilation of myelin sheath was recognized in both the forebrain and cerebellar WM.

#### 3.2.2. Brains Kept at 22 °C

##### T1 (24 h)

**Figure 3:**
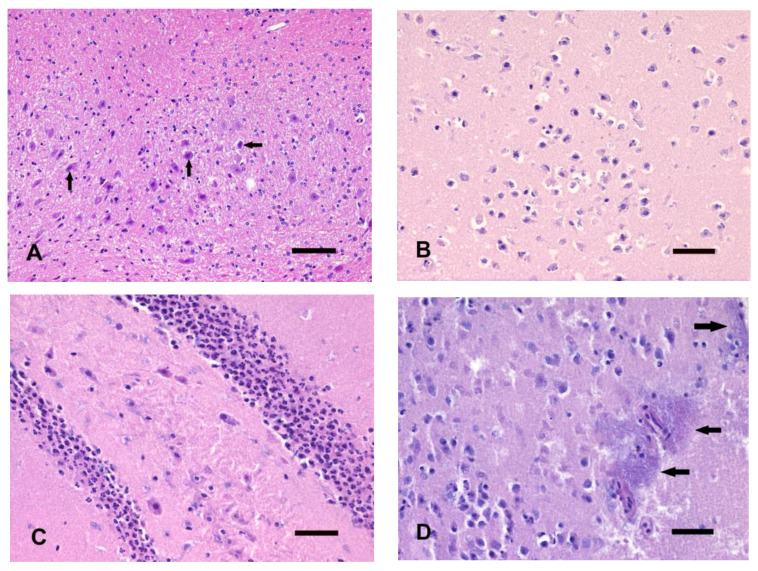
Brains kept at 22 °C. (**A**) T1. Some neurons of the cerebellar nuclei show cytoplasmic hypereosinophilia and pyknosis (arrows), which are associated with microvacuolation of the neuropil. (**B**) T3. Nuclear pyknosis and fragmentation of most thalamic neurons. (**C**) T4. Advanced cell shrinkage, pyknosis and karyorrhexis of the dentate gyrus. (**D**) T4. Pericapillary proliferation of bacteria (arrows) with ghost and karyorrhectic cells. HE, bar = 50 µm.

Less than half of the cortical neurons showed hyperchromatic and often pyknotic nuclei. Cytoplasmic vacuolation was observed in some neurons of the III and V cortical layers. Pyknotic glial nuclei, prevalently oligodendrocytes, were scattered within the neocortex. Neurons of thalamic nuclei showed marked cytoplasmic vacuolation with almost normal nuclei and prominent nucleolus. The dentate gyrus appeared markedly vacuolated, with most neurons showing nuclear pyknosis, whereas karyorrhexis was evident in only a minimal number of neurons. Nuclear hyperchromasia and pyknosis were only occasionally observed in the hippocampal pyramidal cells. Almost 50% of cerebellar granule cell nuclei showed pyknosis, with a few cells showing karyorrhexis. A few Purkinje cells showed cytoplasmic microvacuolation and shrinkage, accompanied by nuclear hyperchromasia, whereas most Purkinje cells remained relatively unchanged. Scattered neurons of the cerebellar nuclei showed hypereosinophilic cytoplasm accompanied by hyperchromatic and pyknotic nuclei (Figure 3A). Based on the morphological changes of the GM, 5 brains were assigned a score of 2 and 2 a score of 1. The WM showed autolytic changes characterized by multifocal vacuolation in all examined areas.

##### T2 (120 h)

An increased number of cortical neurons showed pyknotic nuclei with irregular and spiky nuclear profiles accompanied by pale to hypereosinophilic cytoplasm. Only a few karyorrhectic nuclei were observed. In the thalamic nuclei, almost all neurons displayed cytoplasmic diffuse vacuolation. Nuclear pyknosis of most thalamic neurons was also observed, and some neurons showed different degrees of karyorrhexis, culminating in two cases where only a few punctiform basophilic nuclear fragments were detected. More than half of the neurons of the dentate gyrus showed karyorrhexis and nuclear pyknosis was evident in the remaining cells. In the hippocampus, a numerical reduction of neurons was recorded, accompanied by neurons displaying cytoplasmic vacuolation, sometimes fading, with nuclear pyknosis and only occasional karyorrhexis. In the cerebellum, the granule cells appeared much less compact than at T1, and pyknotic/karyorrhectic nuclei were scattered or barely grouped, surrounded by a pale eosinophilic acellular matrix. Purkinje cells appeared more altered with cytoplasmic and pericellular vacuolation, accompanied by nuclear hyperchromasia of almost all cells. A perineuronal and periglial cell halo with intense vacuolation, WM disarrangement, and axonal fragmentation were widely observed.

##### T3 (168 h)

Most cortical neurons and glial cells showed karyorrhexis. Thalamic nuclei showed similar morphological changes compared to T2 (Figure 3B). All neurons of the dentate gyrus showed karyorrhexis with cellular depletion and residual punctiform basophilic nuclear debris. Pyknosis was observed in more than half of hippocampal pyramidal cells. Areas of cellular depletion in the cerebellar granule cells were evident, with almost all Purkinje cells showing hypereosinophilic vacuolated cytoplasm and nuclear changes, varying from hyperchromasia to irregular nuclear profiles and granular appearance of the nuclei. Some Purkinje cells also displayed clear, perinuclear spaces. Autolytic changes were more evident in the deep cerebellar WM and folia than in forebrain WM.

##### T4 (336 h)

Putrefactive gas bubbles were prevalently located in the superficial cortical layers. More than half of cortical neurons showed pale cytoplasm with barely distinguishable cytoplasmic borders and disappeared nuclei (“ghost-like” appearance); the remaining neurons showed advanced karyorrhexis. Cytoplasmic dissolution with only residual hypereosinophilic material and karyorrhectic debris was observed in the thalamic nuclei of all brains, accompanied by marked perivascular vacuolation. Intravascular and perivascular accumulation of rod-shaped bacteria was detected in the neuroparenchyma and in the leptomeninges of the ventral thalamic area. CA3 neurons showed cellular homogenization with loss of discernible nuclei, indistinct cell borders, and only scattered pyknotic nuclei. In the other areas of the Ammon’s horn, advanced nuclear pyknosis was observed. Karyorrhectic debris was evident as remnants of dentate gyrus neurons (Figure 3C). Dissolution of cerebellar granule cells prevalently at the tip of the folia was evident. Areas devoid of granule cells were observed prevalently in the cerebellar sulci, where only punctiform basophilic nuclear remnants were detected. Purkinje cells often showed cytoplasmic homogenization with uniformly eosinophilic or hypereosinophilic cytoplasm with loss of nuclear details. A “ghost-like” appearance was evident in several Purkinje cells. Putrefactive gas bubbles were observed in the cerebellar cortex and inconsistently in the cerebellar WM. Bacteria were prevalently located within the lumen of leptomeningeal and neuroparenchymal blood vessels and spread perivascularly into the surrounding neuroparenchyma and meningeal space (Figure 3D). A complete disarrangement of WM bundles and a marked reduction of LFB stainability were evident.

#### 3.2.3. Brains Kept at 37 °C

##### T1 (24 h)

**Figure 4:**
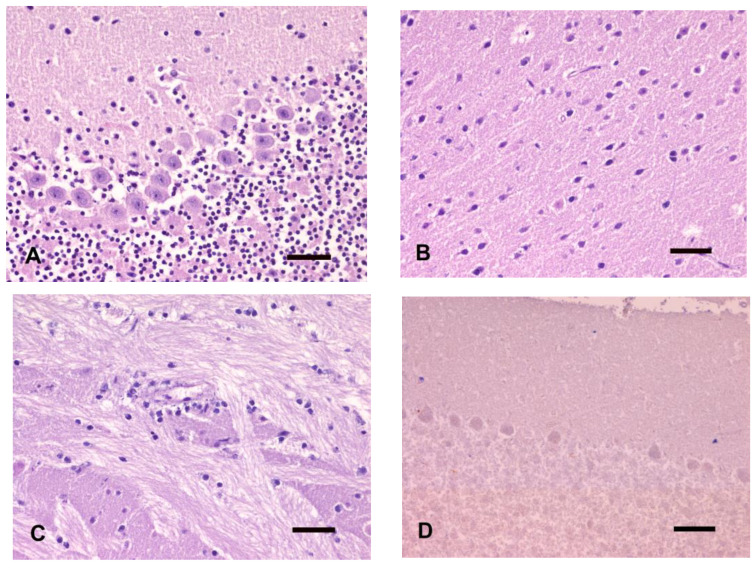
Brains kept at 37 °C. (**A**) T1. Cerebellar cortex. Microvacuolation of molecular and granule cell layers and pyknotic microneurons. (**B**) T2. Cerebral cortex. Shrinkage of cortical neurons and nuclear pyknosis. (**C**) T3. Internal capsule. Severe myelin dilation and nuclear debris. (**D**) T4. Cerebellar cortex. Pale tissue staining with ghost cells and scattered basophilic debris. HE, bar = 50 µm.

Nuclear hyperchromasia of cortical neurons and thalamus was observed, and some pyramidal cortical neurons also displayed hypereosinophilic cytoplasm. Perineuronal and perivascular clear halos were evident in all examined areas. In the hippocampus, advanced pyknosis and karyorrhexis involved almost all neurons of the dentate gyrus, whereas pyramidal neurons displayed only nuclear pyknosis. In the cerebellar cortex, pyknosis of several granule cells and diffuse microvacuolation of molecular and granule cell layers was observed (Figure 4A). Similar morphological changes also involved the deep cerebellar nuclei area. Based on the degree of the autolytic changes, an overall score of 2 was assigned to the GM of all cases. The WM of all examined brains showed moderate to severe dilation of myelin sheath and fragmentation, accompanied by pyknotic glial cells.

##### T2 (120 h)

Diffuse shrinkage of cortical neurons and nuclear pyknosis, accompanied by a moderate amount of karyorrhectic debris, were detected (Figure 4B). Putrefactive gas bubbles were scattered and sometimes grouped prevalently in the superficial layer of the neocortex. Diffuse karyorrhexis involved thalamic neurons. In the hippocampus, almost all pyramidal neurons showed hypereosinophilic cytoplasm, and neurons of the caudal area displayed a “ghost-like” appearance, accompanied by advanced karyorrhexis of neurons of the dentate gyrus. Marked karyorrhexis of the cerebellar granule cells with clear areas of dissolution and neuronal loss were evident. Putrefactive bubbles were detected prevalently in the superficial layer of the cerebellar folia with destruction of the neuroparenchyma. Bacteria were recognized prevalently within the lumen of leptomeningeal vessels. Almost all Purkinje cells displayed hypereosinophilic cytoplasm with barely distinct nuclei and almost only a discernible centrally located nucleolus. Similar changes also involved the neurons of the cerebellar nuclei. A score of 1 was assigned to all brains due to the prevalence of nuclear changes (pyknosis and karyorrhexis). In the WM, dilation and fragmentation of the myelin sheath with reduced affinity for LFB staining were observed. Additionally, the loss of cell borders of the choroid plexus cells, which appeared diffusely hypereosinophilic and multifocally vacuolated, was observed. Erythrocytes were no longer recognizable within the lumen of intraparenchymal vessels and choroid plexus.

##### T3 (168 h)

Partial or complete loss of hematoxylin and Nissl staining affinity of neurons was evident, whereas basophilic dark purple staining was maintained by glial cells. Neurons of the neocortex, thalamus, and hippocampus showed a “ghost-like” appearance and pyknotic and karyorrhectic glial cells were detected in both the GM and WM. Granule cell dissolution was associated with areas completely devoid of cells; only punctiform basophilic nuclear remnants were detected. Almost all Purkinje cells showed a “ghost-like” appearance and only a few were diffusely hypereosinophilic. Pyknotic and karyorrhectic glial cells were scattered in the cerebral (Figure 4C) and cerebellar WM. All brain areas were assigned an overall score of 0.

##### T4 (336 h)

All HE-stained brain sections showed diffuse pale pink staining with the occasional presence of only a few karyorrhectic glial cell debris (Figure 4D); the bacteria were still recognizable. An overall score of 0 was assigned to every brain area.

### 3.3. Immunohistochemistry

The immunoreactivity pattern of all markers was similar in the brain tissue of both control groups (fT0 and pT0). The immunohistochemical scores of experimental groups are summarized in Table 5.

#### 3.3.1. NeuN

In both fT0 and pT0 brains, NeuN expression was localized to the neuronal nuclei, representing the normal immunohistochemical pattern of this marker. The statistical analysis of NeuN immunolabeling between fT0 and pT0 brains revealed no significant differences.

**Figure 5:**
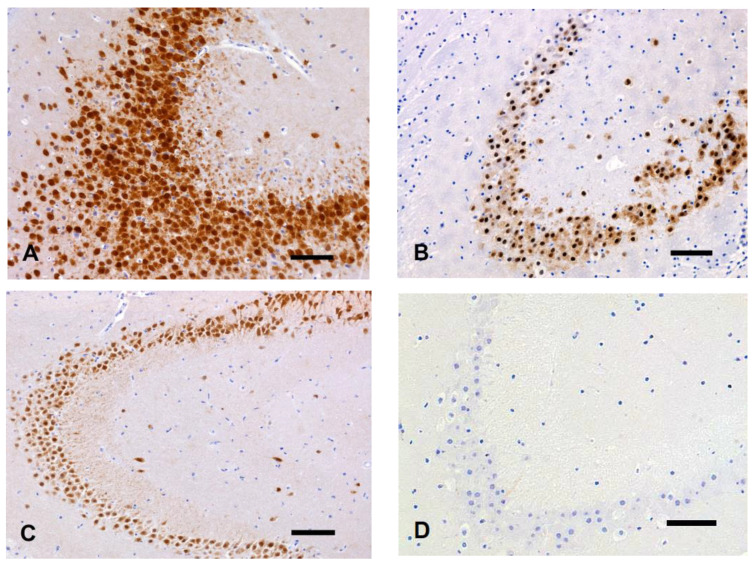
Hippocampus. (**A**) T1 brain kept at 4 °C. Strong immunolabeling of the neuron nuclei of the dentate gyrus. (**B**) T2 brain kept at 4 °C. Diffusion of the immunolabeling to the cytoplasm. (**C**) T2 brain kept at 37 °C. Numerical reduction of immunolabeled neurons. (**D**) T4 brain kept at 4 °C. Complete loss of nuclear immunoreactivity. IHC with anti-NeuN antibody, bar = 50 µm.

In brains kept at 4 °C, immunoreactivity at T1 was predominantly associated with the neuronal nuclei, similar to control tissue (Figure 5A). At T2, a background staining and diffusion of the immunolabeling to the cytoplasm became evident (Figure 5B), and at T3, only some cerebrocortical neurons showed a spiky cytoplasmic profile. A highly significant difference between T1 and T2 was observed in hippocampal cell count (*p* = 0.001). The complete lack of immunoreactivity was restricted to the hippocampus at T4.

A spiky appearance of cerebrocortical neurons was found in brains kept at 22 °C and at 37 °C as early as 24 h postmortem (T1), whereas thalamic neurons maintained a roundish profile. The cerebellar granule cells showed a diffuse, granular and brownish background labeling, although several neurons maintained clear, well-detectable, and immunoreactive nuclei. In brains kept at 22 °C with a delayed fixation of 120 h (T2), a loss of definition of nuclear staining was detected in all areas, and the cell counts were significantly different in the neocortex (*p* = 0.026). At T3, only the hippocampal neurons were consistently immunonegative, and at T4, no immunoreactivity was observed in all areas.

Neurons of brains kept at 37 °C and fixed after 120 h (T2) showed overall reduced immunostaining intensity (Figure 5C). In the thalamus, the cell counts were significantly different compared to T1 (*p* = 0.039). At T3 and T4, no immunoreactivity was observed in all areas (Figure 5D).

#### 3.3.2. SMI-32

**Figure 6:**
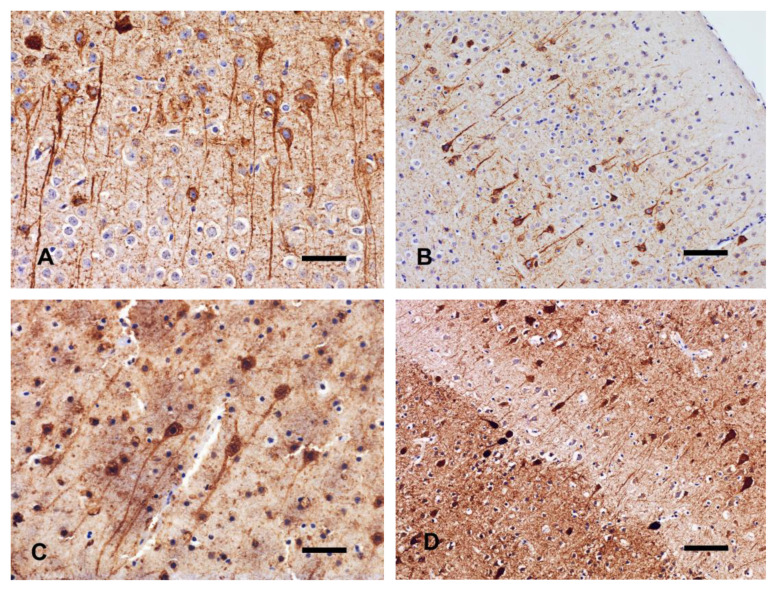
Cerebral cortex. (**A**) T1 brain kept at 4 °C. Normal neurofilament expression in cytoplasm and axons of pyramidal neurons. (**B**) T2 brain kept at 4 °C. Cortical pyramidal neurons and processes are immunoreactive without significant background staining. (**C**) T3 brain kept at 22 °C. Immunolabeling of pyramidal and smaller cortical neurons accompanied by diffuse background granular staining. (**D**) T4 brain kept at 37 °C Strong laminar background staining with immunoreactive shrunken cells. IHC with anti-SMI-32 antibody, bar = 50 µm.

Brains kept at 4 °C did not show significant differences compared to control brains at T1 (Figure 6A), having a similar SMI-32 immunolabeling intensity and pattern. At T2, T3, and T4, immunolabeled axons were detected prevalently in the cerebellar WM, internal capsule, and corpus callosum, accompanied by immunolabeling of scattered thalamic and cerebellar nuclei neurons. The immunolabeling of the neocortex remained restricted to large pyramidal neurons with no detectable background staining (Figure 6B).

Marked and diffuse SMI-32 immunolabeling was detected in both brains kept at 22 °C and 37 °C, starting as early as T1, and progressively increased to T4 with more severe changes in specificity in brains kept at 37 °C. Immunolabeling of cerebrocortical and thalamic neurons, Purkinje cells, cerebellar nuclei, and axons in the WM was associated with a progressive increase of background staining in tissues subjected to prolonged fixation delay and an increase in temperature (Figure 6C,D).

#### 3.3.3. 2F11

**Figure 7:**
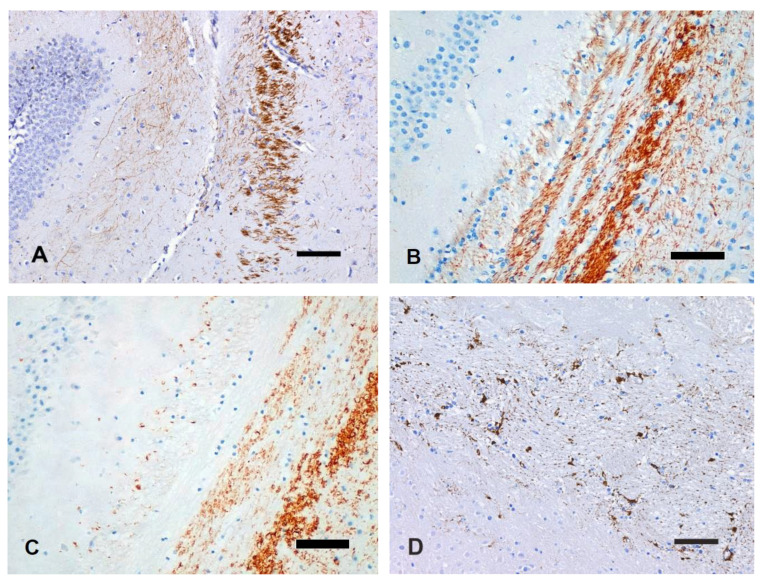
The section at the level of the hippocampus and internal capsule. (**A**) fT0 brain. Normal axons express phosphorylated neurofilaments. (**B**) T1 brain kept at 4 °C. Intensity and specificity of the immunoreactivity is comparable to control. (**C**) T4 brain kept at 4 °C. Reduced number of immunolabeled axons with the granular appearance of immunolabeled axonal fragments. (**D**) Immunoreactive residual axonal fragments. IHC with anti-2F11 antibody, bar = 100 µm (**A**), bar = 50 µm (**B**–**D**).

Compared to control tissue, brains kept at 4 °C did not show differences in axon immunolabeling at T1 (Figure 7A,B). At T2, axonal fragmentation was evident in all areas, with a strong immunoreactivity of axons in the internal capsule and cerebellar WM. At T3, a granular appearance of immunolabeled axonal fragments in the cerebral and cerebellar WM was accompanied by the diffusion of a brownish background prevalently involving the cerebellum. At T4, the immunolabeling pattern was similar to T3, although a reduced number of immunolabeled axons was evident in the internal capsule and cerebral peduncles (Figure 7C).

2F11 axonal immunoreactivity of brains kept at 22 °C was slightly modified already at T1. An increase in immunoreactivity was evident with slightly stronger immunolabeling of dilated and fragmented axons. At T2, scattered immunolabeled axon fragments were evident in the cerebellar WM and cerebral peduncles. Similar findings were observed at T3 (Figure 7D). At T4, immunolabeling of axons was completely lacking.

In brains kept at 37 °C, already at T1, only scattered immunolabeled axon fragments were evident prevalently in the cerebellar WM and, beginning at T2, 2F11 immunolabeling was completely lacking in all brain areas.

#### 3.3.4. Olig2

**Figure 8:**
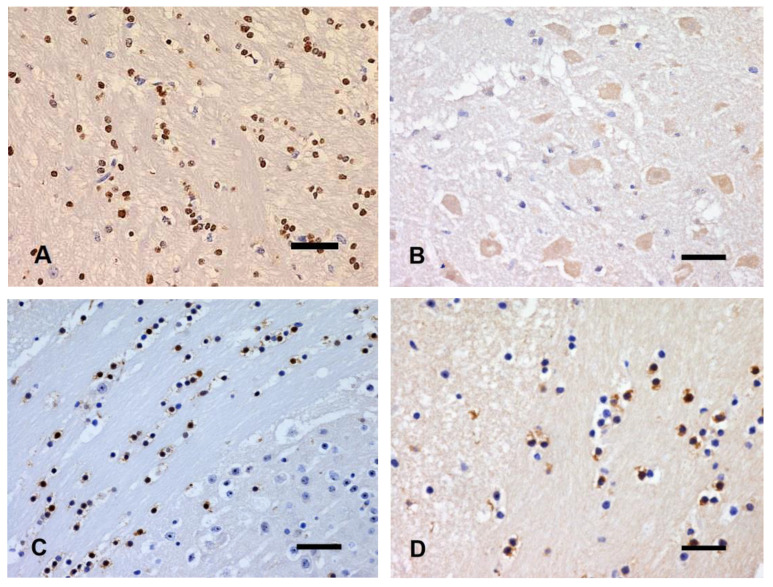
(**A**) Corpus callosum, fT0 brain. Oligodendrocyte nuclei express Olig2. (**B**) Cerebellar nuclei, T4 brain at 4 °C. Oligodendrocyte nuclei are not immunoreactive and frequently karyorrhectic. Neurons are nonspecifically immunolabeled. (**C**) Corpus callosum, T1 brain at 22 °C. Diffusion of the immunolabeling into the cytoplasm of almost all oligodendrocytes. (**D**) Corpus callosum, T2 brain at 22 °C. Loss of immunolabeling of approximately half of oligodendrocytes. IHC with anti-Olig2 antibody, bar = 50 µm.

Oligodendrocyte immunolabeling of brains kept at 4 °C did not differ from control brains at T1 and T2, displaying a dark brown, roundish nuclear profile (Figure 8A). At T3, a slight diffusion of immunolabeling into the cytoplasm, accompanied by irregular nuclear profiles, was prevalently observed in the forebrain WM and occasionally in the cerebellar WM. At T4, almost all oligodendrocytes showed irregular and spiky nuclear profiles with no immunoreactivity, accompanied by very light brown labeling of neurons of the cerebellar nuclei and Purkinje cells, interpreted as antibody nonspecific reaction (Figure 8B).

In brains kept at 22 °C, clearly discernable immunolabeled oligodendrocyte nuclei were observed at T1, comparable to control tissue. Only slight diffusion of immunolabeling into the cytoplasm was evident, prevalently in the corpus callosum (Figure 8C). At T2, a partial loss of nuclear immunoreactivity spreading into the cytoplasm was evident, accompanied by a numerical reduction of immunolabeled oligodendrocytes showing irregular profiles (Figure 8D). At T3, only remnants of scattered immunolabeled pyknotic nuclei and granular immunolabeling of sparse cytoplasmic fragments were observed in all examined areas. At T4, a complete loss of oligodendrocyte immunoreactivity was evident in all brain areas.

Brains kept at 37 °C showed immunolabeling of oligodendrocyte nuclei at T1 with light brown, granular immunostaining diffusion into the cytoplasm. At T2, only a few oligodendrocyte nuclei were still immunolabeled and recognizable, with a nonspecific reactivity of some immunolabeled neurons of the cerebellar nuclei. At T3 and T4, no immunolabeled oligodendrocytes were detected, and a slight nonspecific reactivity of the perikarya of the cerebellar nuclei was still evident, accompanied by some brownish halos scattered in the WM.

#### 3.3.5. GFAP

**Figure 9:**
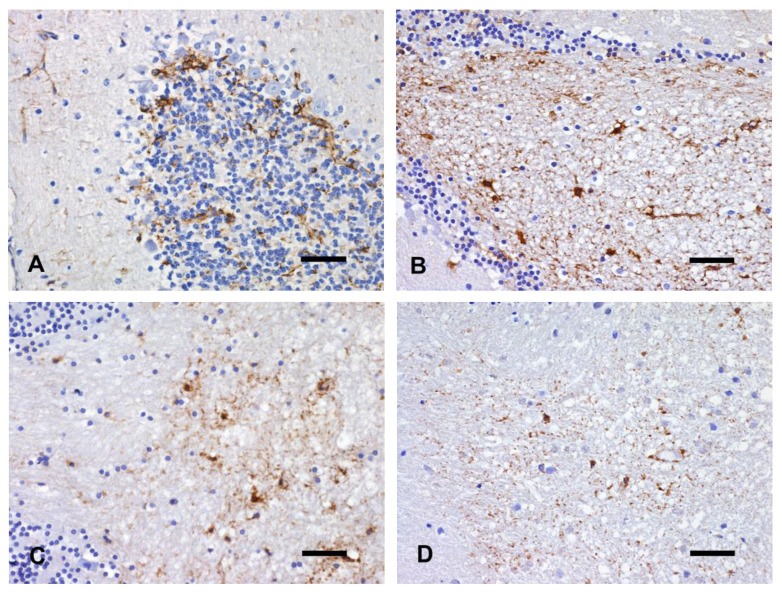
Cerebellum. (**A**) fT0 brain. Normal GFAP expression in the cerebellar cortex. (**B**) T2 brain kept at 4 °C. Recognizable astrocytes and processes within vacuolated WM and granule cell layer. (**C**) T3 brain kept at 22 °C. Fragmentation of astrocytic processes in the WM and disappearance in the granular cell layer. (**D**) T3 brain kept at 37 °C. Reduction of immunostaining intensity of astrocytic cell bodies and residual astrocytic processes. IHC with anti-GFAP antibody, bar = 50 µm.

In brains kept at 4 °C, no differences were found in GFAP immunoreactivity at T1 compared to the control group (Figure 9A). At T2, a granular light brown appearance of the neuropil in the forebrain and cerebellum was observed. Astrocytic cell bodies and processes were still recognizable (Figure 9B). No differences were recorded between T3 and T4. Well-stained dense aggregates of immunolabeled fragments were observed in the internal capsule, thalamus, and cerebellar WM and were accompanied by still well-recognizable immunolabeled astrocytic cell bodies.

Immunoreactivity of astrocytes labeled with GFAP was different already after 24 h (T1) in brains kept at 22 °C and 37 °C. Fragments of astrocytic processes were dispersed throughout the neuropil, and the immunolabeling of the astrocytic cytoplasm was stronger than in control cases, and it was accompanied by the highlighting of short cytoplasmic processes. Perivascular astrocytes showed strong immunoreactivity. In the brains of the T2 group kept at 22 °C, a few discernible astrocytic nuclei were associated with astrocytic fragments dispersed in the cerebral WM. Persistent positivity of subpial astrocytes was also evident. At T3, some round to ovoid immunolabeled fragments, interpreted as remnants of astrocytic cytoplasm, were multifocally distributed in both the internal capsule, corpus callosum, and cerebellar WM (Figure 9C). Strong immunolabeling, although inconsistent, was restricted to subpial and perivascular astrocytes.

Brains kept at 37 °C showed similar features, but in the T2 and T3 groups, a weaker immunoreactivity was detected. Only tiny astrocytic fragments, as well as perivascular and subpial astrocytes, were slightly immunolabeled. Multifocal dense aggregates of immunolabeled fragments were observed in the internal capsule and thalamus. In the cerebellar WM, the reduction in immunoreactivity of almost all astrocytes was accompanied by tiny astrocytic fragments (Figure 9D).

Both brain groups kept at 22 °C and 37 °C with 336 h of delayed fixation (T4), showed complete loss of immunoreactivity in the cerebellum but not in the pons and medulla oblongata in which dispersed fragments were observed in the subpial neuroparenchyma and surrounding the ependymal layer. In the forebrain, residual immunoreactivity of astrocytic fragments was mainly scattered in the internal capsule. Other WM structures did not show any immunolabeled cells.

## 4. Discussion

This study provided insight into the timeline of autolysis in mouse brain tissue at varying storage temperatures over a two-week period. Using a comprehensive approach combining morphological assessments with immunohistochemical analyses, this research aimed to contribute to the understanding of post-mortem changes, which are often overlooked in both forensic science and clinical neurobiology. Although other factors, including variation across species, age, and premortem condition, may affect the rate of postmortem decomposition [21], our results demonstrated that the onset and the progression of autolytic changes varied significantly with temperature and anatomical region. The difference in morphology and antigen expression of nervous tissue between decapitated and whole mice was not appreciable, and therefore, our results can likely be applied to a forensic context.

### 4.1. Histopathology

Histological analysis revealed that autolytic changes began at different time points, with brain samples stored at 22 °C and 37 °C exhibiting early changes, while those kept at 4 °C showed delayed autolysis. In brains stored at 22 °C, only a few cerebellar nuclei neurons retained their Nissl substance, similar to the finding of fragmented remnants of Nissl bodies seen 23.5 h after death at room temperature in a study on guinea pigs [27]. In contrast, a histological and ultrastructural study on rat cortical neurons found no notable changes within 24 h after death at room temperature [19].

Intracellular vacuolization is a characteristic ultrastructural finding in postmortem brain cells, and the number of vacuoles has been shown to increase during the PMI as a consequence of the degradation of cellular compartments [28]. Vacuoles can also be seen in the neuropil during the PMI due to extensive swelling of astrocytic processes and presynaptic terminals [29].

In our study, the WM appeared to be better preserved than GM, which is in agreement with previous results obtained in human and rat tissue [23,30]. Vacuoles in the WM can result from swollen astrocyte processes, swollen axons, or the separation of the myelin sheath from the axon, with fluid filling the resulting space [31].

Different degrees of autolysis were noticed in different brain areas. The cerebellum displayed earlier postmortem changes compared to the forebrain, with the cerebellar granule cells exhibiting considerable vulnerability to autolysis in both brains kept at 22 °C, as already reported [20], and at 37 °C. In human beings and cattle, conglutination of the cerebellar granular cell layer (or “état glace”) occurs due to tissue acidity that is directly related to the increased activity of naphthylamidase, a proteolytic enzyme release at acidic pH (5.5–5.8) by lysosomes in the cerebellar granular cell layer 100 h after death [32,33].

Our findings in the hippocampus revealed early cellular postmortem alterations, supporting the results of previous studies. The hippocampus has been shown to be particularly vulnerable to oxidative stress, arising from high oxidative activity coupled with a relatively low antioxidant content. In particular, the CA1 and CA3 regions of the hippocampus and the dentate gyrus were identified as especially sensitive to oxidative damage [34,35,36]. Postmortem metabolic alterations in rat tissues have been reported to occur as early as 2 h after death, with histological changes becoming prominent at 5 h postmortem [37]. Similarly, Shibayama and Kitoh observed autolytic changes in the rat hippocampus starting from 2 to 3 h after death and increasing at 5 h postmortem [18]. Our study confirmed early postmortem changes in the hippocampus of brains kept at 22 °C and 37 °C, with the dentate gyrus and Ammon’s horn displaying cytoplasmic and nuclear alterations as early as 24 h.

Taking these results together, it emerged that the onset and progression of postmortem autolytic changes of the CNS were related to both postmortem fixation delay and temperature, as already suggested by other histological studies on rats [15,38]. Our findings confirmed the role of the environmental conditions on the onset and rapidity of progression of autolytic changes, as they were as rapid and severe as the temperature increased. Temperature is known to be an environmental factor that influences the autolysis and decomposition of human and animal corpses [39,40,41,42].

### 4.2. Immunohistochemistry

Based on our findings, antigens were differently affected by the autolytic processes, resulting in different alterations, both in terms of specificity and intensity of labeling during the postmortem fixation delay at different temperatures. Similar postmortem alterations in the antigen immunoreactivity followed the same progression in all brains, although being more rapid in brains kept at 37 °C than in brains kept at 22 °C and delayed in brains kept at 4 °C. These results could indicate that antigens are much less affected by temperature than by postmortem delay.

Changes in NeuN immunoreactivity were similar in all experimental groups, starting as early as 24 h postmortem and being more extended and severe in the group of brains kept at 37 °C. The immunohistochemical pattern was characterized by a gradual loss of nuclear immunoreactivity and loss of nuclear definition with a diffusion of the immunolabeling to the cytoplasm probably related to nuclear changes (i.e., pyknosis) already observed in HE sections. The diffusion of the immunolabeling into the cytoplasm could be linked to subtle alterations of the nuclear membrane due to autolysis since this phenomenon became more widely distributed as the postmortem delay increased. However, a perinuclear cytoplasmic localization of NeuN protein has been reported in most normal neurons of mammal CNS [43]. Sarnat and colleagues reported that NeuN immunoreactivity tends to degrade within 6 or 12 h of PMI [44]. Different findings were observed by Koehler and colleagues [26], who did not describe NeuN immunolabeling decrease in canine brain tissue, except after 72 h of fixation delay in brains kept at 37 °C where no discernable labeling could be observed because of total collapse of neuronal structural integrity.

The trend in immunostaining changes has also been confirmed by NeuN-positive cell counting. The difference in cell counts between control brains and brains kept at 22 °C and 37 °C was statistically significant, whereas, at 4 °C, only the difference in hippocampal cell counts at T2 was significant when compared to T1 and control tissues. Similar findings were reported in a study [37], in which a numerical reduction of immunolabeled neurons was observed as early as 30 min and up to 5 h postmortem in the hippocampus of mice.

A strong sensitivity with gradual loss of specificity was observed within different groups using anti-SMI32 antibodies. These findings differed from those of Hilbig and colleagues [22], who detected SMI-32 immunolabeled neurons until a postmortem delay of 12 h in brains kept at 4 °C and 22 °C, following which their presence decreased and only a granular-like staining of dendritic debris was reported. Those differences could be related to the use of a different clone since different epitopes of the same protein can be variably affected by the PMI [45]. Neurofilament cleavage due to the autolytic process, together with the activity of phosphatases, could also lead to the unmasking of axonal and neuronal antigenic epitopes, resulting in enhanced immunolabeling of remaining cellular components. Background enhancement and increase of false positive results have been reported in autolytic skin tissue [46].

The intensity of 2F11 immunoreactivity showed no significant differences in brains kept at 4 °C, whereas a progressive decrease in immunolabeled axons was observed in brains kept at 22 °C and 37 °C. The decrease in 2F11 immunoreactivity could be interpreted as the result of progressive postmortem dephosphorylation of axonal neurofilament proteins. Indeed, it has been shown that the PMI and the storage temperature of the nervous tissue have an important impact on the levels of proteins in the phosphorylated state [47].

Immunolabelling diffusion was observed with the Olig2 antibody. A granular light cytoplasmic immunoreactivity was detected in brains kept at 37 °C and at 22 °C already at T1. The cytoplasmic diffusion and the loss of nuclear immunolabeling were probably related to early nuclear autolytic changes (i.e., pyknosis and karyorrhexis) observed in HE-stained oligodendrocytes. Unexpectedly, a slight immunoreactivity of some neurons in the cerebellar nuclei and Purkinje cells was observed at T3 and T4, interpreted as an antibody nonspecific reaction probably due to the unmasking of some epitopes in the autolytic neuroparenchyma. Similar findings were reported by Koehler and colleagues, where Olig2 immunolabeling was less confined to the nucleus with increasing autolysis [26].

An increased intensity of GFAP immunolabeling of astrocytic cytoplasm and perivascular astrocytes was detected already at T1 in brains kept at 22 °C and 37 °C. Multiple studies reported that postmortem proteolysis leads to more accessible epitopes of GFAP [22,48]. Hilbig and colleagues observed that GFAP immunoreactivity in brains stored more than 8 h was restricted to the pial surface, and stable GFAP-immunolabeled glial structures of the corpus callosum were evident after 24-h postmortem delay [22]. In our study, ovoidal immunolabeled fragments, presumably rims of astrocytic cytoplasm, were still weakly immunolabeled at 168 h postmortem (T3) and the corpus callosum was early affected by autolytic changes. In another study [20], it was reported that GFAP immunolabeling of astrocytes in the cerebellar WM persisted for 3 weeks, whereas in our study, the reduction of immunoreactivity was more pronounced in the cerebellar GM. Conversely, Koehler and colleagues reported no loss of GFAP immunolabeling intensity at any time but a loss of specificity due to advanced autolysis [26].

## 5. Conclusions

This research demonstrated how analysis of the morphology of nervous tissue combined with an IHC panel, including markers of its different components, can help establish the PMI. Morphological stains are more resistant to changes in the PMI because they label classes of biomolecules rather than specific ones. On the other hand, the interpretation of IHC signals for unstable antigens can be challenging, but it is useful in interpreting PMI.

The cerebellum and hippocampus were identified as the two brain regions that were impacted by postmortem changes as early as 24 h. Brains stored at 4 °C displayed autolytic alterations only after 120 h; therefore, determining the PMI up to 5 days could be challenging in animals discovered deceased in cold environmental conditions. NeuN has been proven to be a useful marker in the differentiation between 120 h and a less delayed fixation time at 4 °C when assessed in the hippocampus.

Further research comparing how PMI affects the CNS in various animal models, including larger animal species where the deeper portion of the brain may have a different temperature than the outermost portion, is needed to determine how closely postmortem autolytic changes can be compared in order to offer valuable insights for the analysis of forensic cases.

## Figures and Tables

**Table 1:** The morphological scoring system of grey and white matter.

	Score	Histopathological Features
Grey matter	0	cellular homogenization (“ghost-like” appearance)
	1	cytoplasmic hypereosinophilia; nuclear pyknosis/karyorrhexis
	2	cytoplasmic vacuolation/shrinkage; nuclear hyperchromasia
	3	no morphological abnormalities
White matter	0	severe dilation and fragmentation of the myelin sheath; severe loss of staining intensity
	1	moderate dilation and fragmentation of the myelin sheath; moderate loss of staining intensity
	2	mild dilation of the myelin sheath
	3	no morphological abnormalities

**Table 2:** Primary antibodies used for immunohistochemistry.

Antigen	Antibody	Antigen Retrieval	Dilution	Source
NeuN	mouse mAb ^1^	HIER ^3^	1:1400	Millipore, Burlington, MA, USA
SMI32	mouse mAb ^1^	HIER ^3^	1:100	Millipore, Burlington, MA, USA
2F11	mouse mAb ^1^	HIER ^3^	1:1500	Dako, Glostrup, Denmark
GFAP	rabbit pAb ^2^	NT ^4^	1:1000	Dako, Glostrup, Denmark
Olig 2	rabbit pAb ^2^	HIER ^3^	1:500	Millipore, Burlington, MA, USA

^1^ mAb, monoclonal antibody; ^2^ pAb, polyclonal antibody; ^3^ HIER, heat-induced epitope retrieval with Tris EDTA pH 9; ^4^ NT, no treatment.

**Table 3:** Scoring system for immunohistochemistry evaluation. Features of each experimental group are compared to control groups.

	Score	Immunohistochemical Features
Specificity	0	labeling of non-target cells and structures
	1	consistent changes in the immunostaining pattern
	2	slight changes in the immunostaining pattern
	3	expected immunostaining pattern
Intensity	0	very faint or absent immunostaining
	1	immunostaining lighter than control group
	2	immunostaining intensity similar to control group
	3	immunostaining stronger than control group

**Table 4:** Morphological scores of both grey (GM) and white matter (WM) at different temperatures and time points.

Temperature	Time Points (h)	Score	
		GM	WM
4 °C	T1 (24)	3	3
	T2 (120)	2	3
	T3 (168)	1	2
	T4 (336)	1	2
22 °C	T1 (24)	2	2
	T2 (120)	1	1
	T3 (168)	1	1
	T4 (336)	0	0
37 °C	T1 (24)	2	2
	T2 (120)	1	1
	T3 (168)	0	0
	T4 (336)	0	0

**Table 5:** Intensity and specificity of each immunohistochemical marker at different temperatures and time points.

Temperature	Time Points (h)					Score					
		NeuN		SMI		2F11		Olig2		GFAP	
		I	S	I	S	I	S	I	S	I	S
4 °C	T1 (24)	2	3	2	3	2	3	2	3	2	3
	T2 (120)	2	2	2	2	2	3	2	3	2	2
	T3 (168)	2	2	2	2	2	3	2	2	2	2
	T4 (336)	2	2	2	2	2	3	0	0	2	2
22 °C	T1 (24)	2	2	2	2	2	3	2	2	3	2
	T2 (120)	1	2	2	2	2	3	1	2	2	2
	T3 (168)	1	2	2	2	2	3	1	1	2	1
	T4 (336)	0	0	2	2	0	0	0	0	2	1
37 °C	T1 (24)	2	2	2	2	1	3	2	2	3	2
	T2 (120)	1	2	2	1	0	0	1	1	1	2
	T3 (168)	0	0	2	1	0	0	0	0	1	1
	T4 (336)	0	0	2	0	0	0	0	0	1	1

Legend: I = Intensity; S = specificity.

## Data Availability

Data are contained within the article.
